# Detection of Hodgkin Transformation in a Case of Chronic Lymphocytic Leukemia by PET/CT

**DOI:** 10.4274/mirt.362

**Published:** 2014-06-05

**Authors:** Sabire Yılmaz, Meftune Özhan, Sertaç Asa, M. Sait Sağer, Fatih Selçuk Biricik, Metin Halaç, Kerim Sönmezoğlu

**Affiliations:** 1 Istanbul University Cerrahpasa Faculty of Medicine, Department of Nuclear Medicine, İstanbul, Turkey; 2 İstanbul University Cerrahpaşa Faculty of Medicine, Department of Clinical Oncology, İstanbul, Turkey

**Keywords:** 18F-FDG, Leukemia, lymphocytic, chronic

## Abstract

Richter’s transformation (RT) represents the development of high grade lymphoma, most commonly diffuse large B-cell lymphoma, in patients with chronic lymphocytic leukemia or small lymphocytic lymphoma (CLL/SLL). CLL/SLL may convert also to Hodgkin’s lymphoma, the so-called Hodgkin’s variant of Richter transformation. Histopathological proof is needed to confirm a definitive diagnosis. Patients with RT generally have a poor prognosis, with prompt recognition optimise clinical management. Whole-body PET scan with 18F-FDG can be used for detection of RT of CLL/SLL. We describe the case of 64-year-old woman with CLL/SLL who developed Hodgkin lymphoma detected with PET/CT.

## Abstract (tr)

Richter transformasyonu (RT), kronik lenfositik lösemi veya küçük lenfositik lenfoma (KLL) hastalarında en sık diffuz büyük B-hücreli lenfoma olmak üzere yüksek grade lenfoma gelişmesi demektir. KLL ayrıca Hodgkin varyant Richter transformasyonu olarak bilinen Hodgkin lenfomaya da dönüşebilir. Kesin tanı için histopatolojik kanıt gerekir. RT’li hastalar genellikle kötü prognoza sahiptir. Hastalığın erken tanınması optimum klinik müdahaleyi sağlar. 18F-FDG ile tüm vücut PET sintigrafi RT tespit edilmesinde kullanılabilir. Biz bu olguda, 64 yaşındaki bir KLL hastasında Hodgkin lenfoma gelişiminin PET/BT ile saptanmasını paylaşmak istedik.

## INTRODUCTION

Chronic lymphocytic leukemia (CLL), the most common type of adult leukemia, is characterized by a monoclonal proliferation of mature B-cell lymphocytes with distinctive phenotypic features (1). CLL is a heterogeneous disease with variable prognosis; some patients have an indolent course, while others have aggressive disease and a short survival (1,2). Development of high-grade non-Hodgkin’s lymphoma (NHL) in patients with CLL or SLL is termed as Richter’s transformation (RT) (3). Most commonly, the histology seen in patients with RT is the diffuse large B-cell lymphoma (DLBCL), prolymphocytic leukemia as well as Hodgkin lymphoma (HL) and T-cell lymphoma. (3). The incidence of transformation to DLBCL and HL is approximately 5% and 0.4%, respectively (4,5). 

Although RT involves most frequently the lymph nodes, extranodal localizations such as gastrointestinal tract (4), skin (4), bone marrow (4,6), tonsil (4) may be seen. RT involvement is restricted to single nodal or extranodal lesion in some cases (7). The use of PET in diagnosing and in predicting RT involvement of a given organ is currently under investigation (7). Here, we report the case of a 64-year-old female with CLL/SLL who developed Hodgkin’s lymphoma detected by PET/CT. 

## CASE REPORT

A 64-year-old woman with 7-year history of CLL/SLL was admitted to the hematology clinic with rapidly developing left cervical swelling. She had no history of previous chemotherapy or radiotherapy treatment. On physical examination, she had diffuse palpable adenopathy. Cervical ultrasound demonstrated multiple bilateral lymphadenopathies. Her LDH level was 252 U/L and her routine blood test was normal. She didn’t report any night sweats, fever or weight loss. She underwent a right cervical biopsy. Histopathological examination confirmed the CLL/SLL and she was referred to our unit for reevaluation with FDG PET/CT. 

FDG PET/CT imaging revealed multiple lymph nodes with minimally increased FDG uptake at the right cervical, right supraclavicular, bilateral axillary and various infradiaphragmatic lymphatic stations which were compatible with low FDG uptake of indolent lymphoma. PET/CT images also revealed left submandibular and upper-middle jugular conglomerate lymph nodes with intense FDG uptake (SUVmax was 13.0) suggesting transformation in this patient which was clinically unsuspected before PET/CT study (Figure 1a and 1b). Excisional biopsy of the left cervical lymph node which had maximum FDG uptake was performed and revealed classical Hodgkin’s disease. 

## LITERATURE REVIEW AND DISCUSSION

Histological transformation of indolent lymphoma to a higher grade of lymphoma, most commonly to DLBCL, is typically associated with a poor prognosis. It represents an indication for high dose chemoimmunotherapy with consideration of postremission autologous stem cell transplantation (8). Viral infections may also trigger RT. Ebstein-Bar Virus (EBV) infection has been identified in a few patients with RT and has been implicated in the pathogenesis of some other B-cell malignancies, such as endemic Burkitt lymphoma and Hodgkin’s disease (9,10). 

Although a number of biological and clinical risk factors can be helpful in predicting RT, biopsy of the index lesion is mandatory to make a definitive diagnosis. In one study, lymph node size 3 cm was the sole clinical risk factor of RT selected by two models of multivariate analysis (6). Transformation is suspected if at least one of the following parameters is present rapidly enlarging lymph nodes, increasing LDH level or new onset of B symptom (fever, night sweats, and weight loss greater than 10%) (8). In such cases, PET/CT may be of benefit by depicting the site of transformation (8). The PET characteristics of the lesion, especially the SUV value, help in choosing the biopsy site. PET/CT was also able to recognize clinically unsuspected Richter’s transformation. Bruzzi et al. demonstrated that the overall sensitivity and specificity of PET/CT for RT was 91% and 80%, respectively, with positive and negative predictive values of 53% and 97%, respectively with a SUV_max_ threshold of 5 (7). One of the several explanations of relatively lower specificity and positive predictive value (80% and 53%, respectively) for the diagnosis of Richter’s transformation was the inability of PET/CT to distinguish between RT and other 18F-FDG-avid malignancies (7). Because patients with RT generally have unfavorable prognosis, prompt recognition of RT may improve clinical management. The detection of specific sites of elevated 18F-FDG uptake is predictive of RT and can help direct biopsy strategies. 

## Figures and Tables

**Figure 1a:**
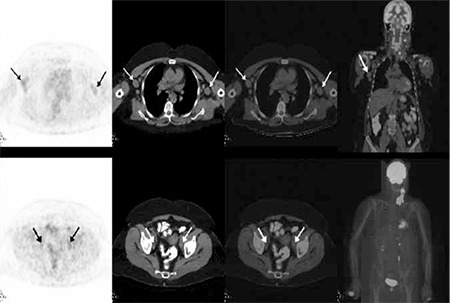
A 64-year-old female patient with CLL/SLL diagnosis. Axial PET, CT, PET/CT fusion and coronal PET/CT fusion images (upper row) of whole-body PET/CT revealed multiple lymph nodes with minimally increased FDG uptake at the right cervical, right supraclavicular, bilateral axillary lymph nodes. There was also minimally increased FDG accumulation at the bilateral internal iliac lypmh nodes (lower row) which was compatible with low FDG uptake of indolent lymphoma.

**Figure 1b:**
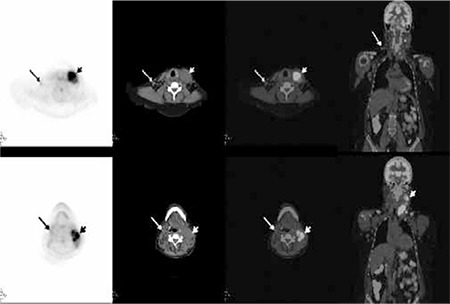
Axial PET, CT, PET/CT fusion and coronal PET/CT fusion images revealed left submandibular and upper-middle jugular conglomerate lymph nodes (short arrow) with intense FDG uptake (SUVmax was 13.0) suggesting transformation. Additionally, minimally increased FDG uptake in the right jugular chain (long arrow) compatible with indolent lymphoma involvement was noted.
